# Cochlear Implantation in Hearing-Impaired Elderly: Clinical Challenges and Opportunities to Optimize Outcome

**DOI:** 10.3389/fnins.2022.887719

**Published:** 2022-07-12

**Authors:** Angelika Illg, Thomas Lenarz

**Affiliations:** Department of Otolaryngology, Hannover Medical School, Hanover, Germany

**Keywords:** cochlear implant, elderly, age, cognitive decline, comorbidities, speech comprehension, MRI, outcome

## Abstract

Cochlear implant (CI) overall provides a very good outcome, but speech comprehension outcome in the elderly is more variable. Several clinical factors play an important role. The management of residual hearing, the presence of comorbidities, and especially the progression of cognitive decline seem to be the clinical parameters that strongly determine the outcome of cochlear implantation and need to be discussed prospectively in the consultation process with the elderly hearing impaired. In the context of this review article, strategies for dealing with these will be discussed. Timely cochlear implantation should already be considered by hearing aid acousticians or practicing otolaryngologists and communicated or initiated with the patient. This requires intensive cooperation between hearing aid acousticians and experts in the clinic. In addition, residual hearing and comorbidities in the elderly need to be considered to make realistic predictions about speech comprehension with CI. Long-term aftercare and its different implementations should be discussed preoperatively, so that the elderly person with hearing impairments feels well taken care of together with his or her relatives. Elderly patients with hearing impairments benefit most from a CI in terms of speech comprehension if there is a large cochlear coverage (electrical or acoustic electrical) and the therapy is not hampered by comorbidities, especially cognitive decline.

## Introduction

The World Report on Hearing determines in the foreword: “Over 1.5 billion people currently experience some degree of hearing loss, which could grow to 2.5 billion by 2050” (World Health Organization). European epidemiological studies have shown that approximately 30% of men and 20% of women have a hearing loss (HL) of at least 30 dB at the age of 70 years. At the age of 80 years, the number of persons with HL further increases to about 55% of men and 45% of women (Roth et al., 2011). It has also been found that the prevalence of comorbidities, e.g., visual impairment, mobility impairment, cognitive impairment, and mental health problems, is higher in people with HL (Besser et al., 2018). Thus, the authors of the Global Burden of Disease Study (GBD, 2016) call for more research investigating a proprietary approach between hearing impairment and comorbidities.

Most cases of HL are sensorineural in origin. For those affected, there is the option of amplifying sound using hearing aids and delivering it to the dysfunctional hair cells or replacing it electrically through direct stimulation using a cochlear implant (CI).

Even at the age over 65 years, which the United Nations defines as the age of onset for elders (United Nations, 2019), CI is still safe and effective, as shown in the literature review by Cosetti and Lalwani (2014). Similarly, cochlear implants can be successfully implanted and used in patients older than 80 years (Williamson et al., 2009; Lenarz et al., 2012).

The variability of patients’ postoperative speech understanding outcomes demonstrates that individual factors determine the degree of benefit from CIs. In this regard, duration of hearing loss, extent of residual hearing, age at onset of deafness, etiology of hearing loss, cognitive decline or other additional disabilities, anatomic cochlear conditions, insertion of electrode array, and number of active electrodes, among others, play a critical role in postoperative speech comprehension with CI (Smulders et al., 2017).

Therefore, it is necessary to address CI and its associated follow-up in the elderly based on the current state of the art. This review article describes the challenges and opportunities in individualized hearing rehabilitation in the elderly with hearing impairment, especially with the clinical perspective of dealing with residual cochlear hearing, the presence of comorbidities, and cognitive decline, as dealing with these factors in the elderly with hearing impairment differs in part from dealing with younger hearing impaired, obviously strongly influencing reintegration into social life.

In the following paragraphs, the scientific status of residual hearing, comorbidities, and cognitive decline will be presented, and then, strategies for individual rehabilitation are derived in the discussion.

## Low-Frequency Residual Hearing in the Elderly and Cochlear Implant

The typical audiological picture of a presbyacusis is the loss of hearing in the high frequencies. A progressing presbyacusis commonly goes along with an increasing high-frequency loss. Patients with high-frequency deafness and residual hearing in the low frequencies under 500 Hz also benefit from CI (James et al., 2005). Specifically, these patients benefit from electric acoustic stimulation (EAS) or, as a synonym, hybrid systems (Büchner et al., 2017). The EAS consists of a speech processor that combines low-frequency acoustic amplification and high-frequency electrical stimulation (Jurawitz et al., 2014). The risk of preservation of the hearing depends significantly on the selected electrode or electrode length (Jurawitz et al., 2014; Lenarz et al., 2019; Iso-Mustajärvi et al., 2020). Longer electrodes are associated with poorer hearing preservation. Bourn et al. (2020) confirm that in elderly patients, preservation of residual hearing is feasible despite concerns about cochlear fragility. Cochlear fragility can be caused by osteoporosis and increased fracture risk (Singh et al., 2018). To date, to our knowledge, there has been only one study examining the extent of hearing preservation with various thin flexible lateral-wall electrodes and the outcome in speech comprehension 12 months after CI in 89 elderly patients over 65 years (Matin et al., 2021). These results demonstrate as well that hearing preservation in elderly patients with CI is feasible 12 months after activation with shorter electrode arrays—despite concerns about increased susceptibility to trauma in these patients. Compared to other studies, the loss of residual hearing was slightly greater in elderly patients, but after a period of 12 months, this difference was no longer observed (Suhling et al., 2016). The authors suspect age-degenerative processes as the cause of the greater hearing loss in the low frequencies (Matin et al., 2021). Speech comprehension also in the elderly is better in patients with deeper insertion angles and wider cochlear coverage. The results with only the longer electrode (FLEX 28) and electric stimulation show significantly better results than speech comprehension with only the shorter electrode (FLEX 20) and electric stimulation (Matin et al., 2021). The hearing thresholds for the EAS indication differ by up to 20 dB between 125 and 500 Hz compared to younger adults. It is calculated that the preoperative limit for EAS provision in elderly patients is 42.5 dB at 250 Hz and 52.5 dB at 500 Hz to achieve sufficient benefit. Therefore, the preoperative score for monosyllables (unaided) should not fall below 32.5% (Matin et al., 2021).

## Outcome and Comorbidities in the Elderly

Numerous previous studies have investigated the hearing outcome of elderly with CI without residual hearing (Labadie et al., 2000; Pasanisi et al., 2003; Chatelin et al., 2004; Sterkers et al., 2004; Lenarz et al., 2012, refer to the summary by Clark et al., 2012). However, these are often studies with small cohorts like the study by Friedland et al. (2010) where conclusions about the influence of age or methodological other differences were compromised. To date, we are aware of only two studies that have specifically examined comorbidities in the elderly. Wilkerson et al. (2017) described a tendency for more comorbidities in elders (>70 years, *n* = 50) with CI compared to younger patients (<69 years, *n* = 51). They listed comorbidities by frequency: hypertension, coronary artery disease, diabetes mellitus type 2, atrial fibrillation, chronic obstructive pulmonary disease, aortic valve stenosis, pulmonary fibrosis, and aortic and cerebral aneurysm each. A correlation of speech comprehension has not been described by the authors, but to the complication rate, which does not differ significantly between younger and older patients even with comorbidities.

Giourgas et al. (2021) examined the effect of CI in 446 elderly patients at the age of 61 to 89 years during the time of unilateral CI (Group 1) with respect to their speech comprehension and compared the data with a randomized group of 110 adults at the age of 17–42 years (Group 2). There was no significant difference between Groups 1 and 2 related to their duration of hearing loss and score for comprehension of monosyllables preoperatively (Group 1: mean 6.6 ± 12.3%; Group 2: mean 5.0 ± 10.6%). Twelve months postoperatively, there was a statistically significant improvement in the monosyllable score (FMT) (Wilcoxon test *z* = −16.41, *p* < 0.001) with a large effect size (*r* = 0.59) of Group 1. The data show that higher age is associated with lower speech comprehension scores. In contrast, there is minimal to no correlation between age and speech perception scores in Group 2 after 1 year of CI experience. The statistical test yielded a difference in the speech comprehension across Groups 1 and 2 for the FMT (*p* = 0.001) but surprisingly, no significant difference for sentences in the noise test (*p* = 0.222). Comorbidities were documented in more than one-third of the patients in Group 1 (37.9%). Multimorbidities have also been documented in several patients (e.g., heart failure and impaired motor skills). The comorbidities were categorized, with cognitive and/or neurological diagnoses forming a separate category. Speech comprehension is most influenced by neurological comorbidities (e.g., condition after apoplectic insult), followed by multimorbidity.

## Cognitive Decline in the Elderly

In recent years, several prospective studies addressed the effects of CI fitting on cognitive function in elderly persons with hearing impairment (e.g., Mosnier et al., 2015; Castiglione et al., 2016; Cosetti et al., 2016; Ambert-Dahan et al., 2017; Jayakody et al., 2017; Sonnet et al., 2017; Claes et al., 2018; Völter et al., 2018; Sarant et al., 2019). The main findings in this regard are listed in the study of Huber et al. (2021). Almost all studies, with the exception of the study by Sonnet et al. (2017), report cognitive improvements after CI fitting. This particularly concerns global cognition (Castiglione et al., 2016; Claes et al., 2018), verbal episodic memory (Cosetti et al., 2016; Claes et al., 2018; Völter et al., 2018), and various areas of executive function (Claes et al., 2018; Völter et al., 2018).

Huber et al. (2021) have studied the central question of whether cognitive changes following CI fitting are large enough to completely reverse cognitive decline due to hearing impairment, back to the levels comparable to normal-hearing participants. To do this, they compared data from participants in an age-matched normal-hearing control group of over 12 months in addition to the patient group with hearing loss. Patients in the normal-hearing control group showed no significant improvement in cognitive performance on any task. The exception was the results in the Stroop test. This tests the ability to suppress unnecessary information and concentrate on essentials. This ability is necessary in communication situations with several people or in noisy environments. The hearing-impaired elderly improved their cognitive performance more than the control group in global cognition measured by the Clock Drawing Test (CDT). The CDT is a test procedure that tests different cognitive abilities together. This mixture refers to semantic memory, fine motor control and planning, visuospatial organization, executive skill planning, control, and coordination of task elements (Eknoyan et al., 2012). The improvement in global cognition was significantly associated with speech comprehension 3 months post-CI, but not with speech comprehension after 12 months. Also, Mosnier et al. (2015) have described significant improvement in CDT values after CI treatment with previously unremarkable cognitive conditions. In the presence of preoperatively conspicuous cognitive findings, no significant improvements have been described after CI treatment. A variety of factors could be considered responsible for a possible cognitive improvement after CI. It has also been known for a long time that patients with hearing impairments suffer from psychological and social problems, e.g., low self-esteem, social activities, and social interaction (Hétu and Getty, 1991). Increased social contact and interaction may be possible through CI and may be a possible reason for the improvement in cognitive abilities. The results after CI treatment in the elderly seem also to depend on their mental health (Knopke et al., 2019). In this study, it is shown that the values of anxiety and depressive symptoms in elderly people between 70 and 88 years correlate with the hearing-related quality of life 1 year after CI. CI use could also contribute to working against the psychological and social deficits of these elderly people. Huber et al. (2021) also describe that the group with hearing loss shows significantly more depressive problems but not more anxiety than their normal hearing peers in the control group. Population studies (Gopinath et al., 2009; Li et al., 2014; Hsu et al., 2016) show that hearing loss in adulthood increases the likelihood of developing depressive problems and disorders. Rutherford et al. (2018) describe potential associations among hearing impairment in the elderly, secondary depressive problems, and cognitive change as follows: hearing loss has negative consequences for neuroplasticity. The chronic overuse of compensatory measures impairs functions of the prefrontal cortex. At the behavioral level, there are impairments in executive functions. In addition, impairments in the prefrontal cortex and the limbic system affect emotional responsiveness, control and regulation of emotions, and processing of emotions, leading to depressive problems. Social isolation and loneliness caused by hearing loss additionally lead to depressive moods. Depressive problems lead to cognitive impairment. Also, physical inactivity, increased frailty, stress, tinnitus, and balance disturbance can lead to depressive problems and cognitive decline. Single studies show a decrease in depressive problems after CI in the elderly (Olze et al., 2011; Choi et al., 2016; Claes et al., 2018). Further studies are necessary to research the context of depressive problems, behavior, loneliness, hearing loss, and cognitive decline.

## Discussion

The success of cochlear implantation is determined by various influencing factors. In the context of this article, the presence of residual hearing and the presence of comorbidities with a special case of cognitive decline are described as influencing obvious clinical factors for elderly patients with hearing impairment with CI to derive strategies for individual decision-making process, hearing rehabilitation, and reintegration into social life. These strategies are necessary for the common decision-making process for CI in the elderly. In elderly with hearing aids, it is known that patients who participate intensively in diagnostic, therapeutic, and counseling appointments are more satisfied with the sound quality and performance of their hearing aids (Convery et al., 2019). The elderly with presbyacusis commonly present a slowly progressive hearing loss, which is treated by the practicing otolaryngologist and acoustician starting with a hearing aid. It is then important to find out the point when conventional hearing aid provision is no longer sufficient for the patient. On the one hand, patients’ satisfaction must be taken into account; on the other hand, the data of speech comprehension and the technical limitations of the hearing aids are to be considered. A network of patient associations, hearing aid acousticians, practicing otolaryngologists, and CI clinics are required for CI care so that the topic of hearing disorders and their care becomes a social focus. In addition, the role of the social environment, such as family and friends, should not be underestimated and must become an object of research. When advising the elderly in particular, the high expectations that go beyond hearing must be taken into account, even if they may not be mentioned (Illg et al., 2021). The areas of cognitive abilities, depression, quality of life, social isolation, or self-management should be considered and addressed from the start. Further studies are needed to clarify more intensively how comprehensive care for elderly patients can be provided with short distances between home and hearing care with the objective of optimal hearing success.

### Strategies for Dealing With Residual Hearing in Elderly Patients With Hearing Impairment—Pros and Cons of Electric Acoustic Stimulation

Up to a moderate hearing loss, a hearing aid fitting provides sufficient amplification of acoustic signals and satisfaction in speech comprehension, which are affected by age, type of hearing impairment, time of daily hearing aid usage, and the threshold of hearing and education (Korkmaz et al., 2016). Regular daily wearing of hearing aids increases satisfaction using the devices. However, a higher hearing loss in the elderly will most likely go along with a deeper dissatisfaction regarding the benefit of hearing aids. Therefore, the question arises as to the ideal time for a CI prescription and how to deal with existing low-frequency residual hearing.

In the case of high-frequency hearing loss, a decreasing benefit of purely acoustic amplification can generally be expected (von Ilberg et al., 2011). Above the limit of 60–80 dB, speech comprehension cannot be further improved by conventional hearing aids. Elderly who suffer from severe sensorineural hearing loss and still have low-pitched residual hearing belong to the group of hearing aid patients who are no longer satisfied with the amplification. The mental transition to CI should always be considered when the patient is no longer satisfied due to poor speech comprehension. Additionally, fears of surgery and electric listening also play a role here and must be taken seriously (Illg et al., 2021). In adults with postlingual deafness, central auditory deprivation has been shown to increase with the duration of deafness in the high frequencies (Sun et al., 2021). In elderly patients with hearing impairment, high-frequency deafness usually begins in adulthood and probably leads to auditory deprivation in the temporal cortex and thalamus. This auditory deprivation affects the postoperative learning process after CI, also in partial hearing loss in high frequencies, which was confirmed by Lenarz et al. (2009). In their study of elderly with amplifiable low-frequency residual hearing but high-frequency deafness, CI users with a shorter duration of high-frequency deafness showed a greater benefit than those with a longer duration of hearing loss. For this reason, timely CI must be considered and discussed with the elderly. The fundamental question here is whether residual hearing can be preserved and used for acoustic amplification even in elderly patients, and how long the residual hearing will be utilizable *via* EAS.

Matin et al. (2021) recommend a very careful selection of EAS use in the elderly. It is necessary to pay particular attention to the preoperative frequencies 250 and 500 Hz. Particularly in the elderly, the case-by-case decision is important, in which the insertion depth must be carefully considered, since the residual hearing deteriorates to a greater extent as a result of the operation than in younger adults and may no longer be usable for acoustic amplification. Skarzynski et al. (2007) point out that the course of progressive hearing loss must be considered individually and should be observed over a period of at least 1 year. If the preoperative residual hearing meets the lower limit of the EAS criteria, long electrodes are the method of choice. When using a long electrode for electric stimulation only, less channel interaction improves spectral resolution, which consequently results in better speech comprehension in noise independent of age (Berenstein et al., 2008; Büchner et al., 2017). However, better perception of music is achieved using EAS (Arnoldner et al., 2010; Brockmeier et al., 2010).

The concept of partial surgical insertion (Lenarz et al., 2019) allows gentle revisions of the electrode placement in case the residual hearing is not preserved during surgery, although there may be a higher risk of complications from anesthesia with multiple revisions (Covert and Fox, 1989; Urwin et al., 2000; Parker et al., 2004). Surgery can be performed under local anesthesia, which avoids the risks and has additional advantages with respect to hearing preservation (Dietz and Lenarz, 2021). In the near future, robot-assisted surgery will facilitate the surgical procedure significantly with shorter duration, surgery under local anesthesia, and on an outpatient basis. Patients can directly react to the changes in their residual hearing as a guidance for electric insertion depth to secure residual hearing. Immediate direct fitting will restore basic communication ability on the day of implantation. The new hearing becomes a routine procedure like cataract surgery.

### Strategies for Dealing With Comorbidities and Cognitive Decline in Elderly Patients With Hearing Impairment

The steady increase in auditory skills during the 1st year of CI use is evident in all elderly patients. However, if the elderly suffer from comorbidities, an influence on postoperative speech comprehension can be suspected, especially if multimorbidities or neurological comorbidities are present. Comorbidities without cognitive or neurological involvement (e.g., heart insufficiency and limited motor skills) also lead to less speech comprehension postoperatively. The more comorbidities an elderly person has, the less the postoperative speech comprehension develops with CI (Giourgas et al., 2021). Knowledge of the individual presence of comorbidities and their treatment, such as medication, as well as knowledge of the influence on postoperative speech understanding is essential in clinical preoperative counseling for a CI. Further studies in elderly patients with CI have to clarify the learning curve in elderly with comorbidities. The speech comprehension test results subjectively indicate that the increase in auditory abilities in the elderly is slower than in the patients at younger ages (Herzog et al., 2003; Lenarz et al., 2012). Otherwise, it is also known that speech comprehension of hearing-impaired patients with CIs shows much less age dependence than that of hearing aid-supplied patients. It is thought that age dependence in hearing aid users is based on the deteriorating hearing threshold (Steffens et al., 2013), which does not change in CI users. Thus, progressive deterioration of speech comprehension in quiet, with intact technology, must be attributed to the presence of comorbidities, alteration of cognitive abilities, or degeneration of the auditory nerve. For example, in the elderly, the speed of internal repetition of sound patterns decreases and a slowing of processing within the central executive arises (Grube, 1999). Latest technical possibilities always influence improvements in speech comprehension (Büchner and Gärtner, 2017) but are not able to balance postoperative speech comprehension results in elderly with comorbidities or cognitive decline.

Cognitive decline is a special case of comorbidity and represents the greatest obstacle to the development of adequate speech comprehension. Cognitive decline manifests itself insidiously and presents a wide variation. Although the patients were not aware of any cognitive impairment, screening results in the routine study of the elderly (Illg et al., 2018) are nevertheless striking in about 35% of the patients. This demonstrates the insidious process that could not be revealed without specific testing in the anamnestic interview alone. Mosnier et al. (2015) also report patients with abnormal cognitive findings in one to three tests of their test battery preoperatively. This leads us to the recommendation to implement cognitive screening in all elderly patients (≥60 years) with hearing impairment preoperatively. Nevertheless, in different age groups, an improvement in the cognitive screening results is already detectable after 3 months of using CI: in the younger elderly age group (65–76 years) earlier than in the older group (>76 years) (Illg et al., 2018). After 12 months, however, the cognitive abilities are not identical to those of normal-hearing peers (Huber et al., 2021).

The “2020 Lancet Commission on dementia prevention, intervention, and care” developed a risk reduction model. In this model, hearing impairment is the main contributor to the development of dementia. It represents 20.5% (8.2 points) of the modifiable factors (Livingston et al., 2020). Sensory deprivation is thought to be an intermediary pathway between hearing loss and cognitive impairment, but the causes have not yet been definitively established (Georgiou, 2020). Strategically, however, the reduction of risk factors is recommended and supported by this commission, as if the causal connection was implied. For example, the use of hearing aids is recommended to halt or slow down hearing loss and prevent or reverse cognitive decline. Caregivers should give importance to a coordinated approach of the different factors. The report lists four additional risk factors that are strongly associated with hearing loss and may be mediated by sensory deprivation: low social contact, depression, traumatic brain injury, and physical inactivity (Georgiou, 2020). It is therefore important to use differential diagnostic test procedures as early as the decision-making phase for a CI, e.g., the Clock Drawing Tests (CDT), Mini-Mental Status Examination (MMSE), or Montreal Cognitive-Assessment (MoCa). These risk factors should also be the focus of further research in elderly patients with profound hearing loss to gain further insights into the development of cognitive decline. In the work with elderly patients with hearing impairment, knowledge about their comorbidities and their risk factors are as important as early CI fitting with optimal individualized technology. Subsequently, combined therapeutic interventions for elderly patients with CI, as well as training auditory and cognitive processes are needed for preventing or stopping dementia.

## Conclusion

The qualified selection of a CI for elderly patients with hearing impairment provides the basis for high-standard CI care and requires special knowledge and handling of this group of persons. A timely CI fitting should be taken into account whenever conventional hearing aids do not show the benefit they should. This requires intensive cooperation between hearing aid acousticians and CI specialists in the clinics. Patient groups can also be an important link in parallel and should offer additional advice and share their personal experiences. Furthermore, the diagnosis and handling of residual hearing and comorbidities, especially cognitive decline in the elderly, is necessary to make realistic predictions about speech comprehension with CI. Long-term aftercare and its different possibilities should be discussed already preoperatively, so that the elderly person with hearing impairment, together with his or her relatives, feels that he or she is in good hands. Elderly patients with hearing impairment benefit most in speech comprehension from a CI when large cochlear coverage (electrical or acoustic electrical) is provided and therapy is not hindered by comorbidities, especially cognitive decline. Further research needs to address borderline outcomes in the elderly with CI to identify additional predictors of success and learning curves in the elderly with and without cognitive decline.

## Author Contributions

AI wrote the manuscript. TL corrected and completed the manuscript. Both authors contributed to the article and approved the submitted version.

## Conflict of Interest

The authors declare that the research was conducted in the absence of any commercial or financial relationships that could be construed as a potential conflict of interest.

## Publisher’s Note

All claims expressed in this article are solely those of the authors and do not necessarily represent those of their affiliated organizations, or those of the publisher, the editors and the reviewers. Any product that may be evaluated in this article, or claim that may be made by its manufacturer, is not guaranteed or endorsed by the publisher.

## References

[B1] Ambert-Dahan E. Routier S. Marot L. Bouccara D. Sterkers O. Ferrary E. (2017). Cognitive Evaluation of Cochlear Implanted Adults Using CODEX and MoCA Screening Tests. *Otol. Neurotol.* 38 e282–e284. 10.1097/MAO.0000000000001464 28806339

[B2] Arnoldner C. Helbig S. Wagenblast J. Baumgartner W. D. Hamzavi J. S. Riss D. (2010). Electric acoustic stimulation in patients with postlingual severe high-frequency hearing loss: clinical experience. *Adv. Otorhinolaryngol.* 67 116–124. 10.1159/000262603 19955728

[B3] Berenstein C. K. Mens L. H. Mulder J. J. Vanpoucke F. J. (2008). Current steering and current focusing in cochlear implants: comparison of monopolar, tripolar, and virtual channel electrode configurations. *Ear Hear.* 29 250–260. 10.1097/aud.0b013e3181645336 18595189

[B4] Besser J. Stropahl M. Urry E. Launer S. (2018). Comorbidities of hearing loss and the implications of multimorbidity for audiological care. *Hear. Res.* 369 3–14. 10.1016/j.heares.2018.06 29941312

[B5] Bourn S. Goldstein M. R. Jacob A. (2020). Hearing Preservation in Elderly Cochlear Implant Recipients. *Otol. Neurotol.* 41 618–624. 10.1097/MAO.0000000000002596 32080030

[B6] Brockmeier S. J. Peterreins M. Lorens A. Vermeire K. Helbig S. Anderson I. (2010). Music perception in electric acoustic stimulation users as assessed by the Mu.S.I.C. test. *Adv. Otorhinolaryngol.* 67 70–80. 10.1159/000262598 19955723

[B7] Büchner A. Gärtner L. (2017). Technische Entwicklungen bei Cochleaimplantaten : stand der Technik [Technical advancements in cochlear implants : state of the art]. *HNO* 65 276–289. 10.1007/s00106-017-0339-7 28303288

[B8] Büchner A. Illg A. Majdani O. Lenarz T. (2017). Investigation of the effect of cochlear implant electrode length on speech comprehension in quiet and noise compared with the results with users of electro-acoustic-stimulation, a retrospective analysis. *PLoS One.* 12:e0174900. 10.1371/journal.pone.0174900 28505158 PMC5432071

[B9] Castiglione A. Benatti A. Velardita C. Favaro D. Padoan E. Severi D. (2016). Aging, cognitive decline and hearing loss: effects of auditory rehabilitation and training with hearing aids and cochlear implants on cognitive function and depression among older adults. *Audiol. Neurootol.* 21 21–28. 10.1159/000448350 27806352

[B10] Chatelin V. Kim E. J. Driscoll C. Larky J. Polite C. Price L. (2004). Cochlear Implant Outcomes in the Elderly. *Otol. Neurotol.* 25 298–301. 15129109 10.1097/00129492-200405000-00017

[B11] Choi J. S. Betz J. Li L. Blake C. R. Sung Y. K. Contrera K. J. (2016). Association of Using Hearing Aids or Cochlear Implants With Changes in Depressive Symptoms in Older Adults. *JAMA Otolaryngol. Head Neck Surg.* 142 652–657. 10.1001/jamaoto.2016.0700 27258813 PMC12861333

[B12] Claes A. J. Van de Heyning P. Gilles A. Van Rompaey V. Mertens G. (2018). Cognitive Performance of Severely Hearing-impaired Older Adults Before and After Cochlear Implantation: preliminary Results of a Prospective, Longitudinal Cohort Study Using the RBANS-H. *Otol. Neurotol.* 39 e765–e773. 10.1097/MAO.0000000000001936 30153132

[B13] Clark J. H. Yeagle J. Arbaje A. I. Lin F. R. Niparko J. K. Francis H. W. (2012). Cochlear implant rehabilitation in older adults: literature review and proposal of a conceptual framework. *J. Am. Geriatr. Soc.* 60 1936–1945. 10.1111/j.1532-5415.2012.04150.x 22974240 PMC3902638

[B14] Convery E. Keidser G. Hickson L. Meyer C. (2019). The Relationship Between Hearing Loss Self-Management and Hearing Aid Benefit and Satisfaction. *Am. J. Audiol.* 28 274–284. 10.1044/2018_AJA-18-0130 31184964

[B15] Cosetti M. K. Lalwani A. K. (2014). Is cochlear Implantation Safe and Effective in Elderly?. *Laryngoscope* 125 1279–1281. 25423907 10.1002/lary.25055

[B16] Cosetti M. K. Pinkston J. B. Flores J. M. Friedmann D. R. Jones C. B. Roland J. T. Jr. (2016). Neurocognitive testing and cochlear implantation: insights into performance in older adults. *Clin. Interv. Aging* 12 603–613. 10.2147/CIA.S100255 27274210 PMC4869653

[B17] Covert C. R. Fox G. S. (1989). Anaesthesia for hip surgery in the elderly. *Can. J. Anaesth.* 36 311–319. 2655951 10.1007/BF03010771

[B18] Dietz A. Lenarz T. (2021). Cochlear implantation under local anesthesia in 117 cases: patients’ subjective experience and outcomes. *Eur. Arch. Otorhinolaryngol.* 279 3379–3385. 10.1007/s00405-021-07061-4 34487218 PMC8419376

[B19] Eknoyan D. Hurley R. A. Taber K. H. (2012). The clock drawing task: common errors and functional neuroanatomy. *J. Neuropsychiatry Clin. Neurosci.* 24 260–265. 10.1176/appi.neuropsych.12070180 23037640

[B20] Friedland D. R. Runge-Samuelson C. Baig H. Jensen J. (2010). Case-control analysis of cochlear implant performance in elderly patients. *Arch. Otolaryngol. Head Neck Surg.* 136 432–438. 20479370 10.1001/archoto.2010.57

[B21] GBD (2016). Disease and Injury Incidence and Prevalence Collaborators. Global, regional, and national incidence, prevalence, and years lived with disability for 328 diseases and injuries for 195 countries, 1990-2016: a systematic analysis for the Global Burden of Disease Study 2016. *Lancet* 390 1211–1259. 10.1016/S0140-6736(17)32154-2 PMC5605509 28919117

[B22] Georgiou A. (2020). *Lancet Commission on Dementia: A Call to Action for Integrated Hearing Healthcare. ENT Audiology News.* Available online at: www.entandaudiologynews.com (accessed February 14, 2022).

[B23] Giourgas A. Durisin M. Lesinski-Schiedat A. Illg A. Lenarz T. (2021). Auditory performance in a group of elderly patients after cochlear implantation. *Europ. Arch. Otorhinolaryngol.* 278 4295–4303. 10.1007/s00405-020-06566-8 33432395 PMC8486703

[B24] Gopinath B. Wang J. J. Schneider J. Burlutsky G. Snowdon J. McMahon C. M. (2009). Depressive symptoms in older adults with hearing impairments: the Blue Mountains Study. *J. Am. Geriatr. Soc.* 57 1306–1308. 10.1111/j.1532-5415.2009.02317 19570163

[B25] Grube D. (1999). *Arbeitsgedächtnis und Zeitverarbeitung im Alter. Pädagogische Psychologie und Entwicklungspsychologie, Band 10.* Münster: Waxmann

[B26] Herzog M. Schon F. Muller J. Knaus C. Scholtz L. Helms J. (2003). Long term results after cochlear implantation in elderly patients. *Laryngorhinootologie* 82 490–493. 12886496 10.1055/s-2003-40896

[B27] Hétu R. Getty L. (1991). Development of a rehabilitation program for people affected with occupational hearing loss 1. A new paradigm. *Int. J. Audiol.* 30 305–316. 10.3109/00206099109072893 1772381

[B28] Hsu W. T. Hsu C. C. Wen M. H. Lin H. C. Tsai H. T. Su P. (2016). Increased risk of depression in patients with acquired sensory hearing loss: a 12-year follow-up study. *Medicine* 95:e5312. 27858911 10.1097/MD.0000000000005312 PMC5591159

[B29] Huber M. Roesch S. Pletzer B. Lukaschyk J. Lesinski-Schiedat A. Illg A. (2021). Can Cochlear Implantation in Older Adults Reverse Cognitive Decline Due to Hearing Loss? *Ear Hear.* 42 1560–1576. 10.1097/AUD.0000000000001049 34028233

[B30] Illg A. Bräcker T. Illg A. Bräcker T. Batsoulis C. Opie J. M. Lesinski-Schiedat A. (2021). CI decision making and expectations by older adults. *Cochlear Implants Int.* 23 139–147. 34963418 10.1080/14670100.2021.2019522

[B31] Illg A. Lesinski-Schiedat A. Bültmann E. (2018). CI-Versorgung bei Senioren auch unter differenzialdiagnostischen Überlegungen. *Sprache Stimme Gehor* 42 24–29. 10.1055/s-0043-118194

[B32] Iso-Mustajärvi M. Sipari S. Löppönen H. Dietz A. (2020). Preservation of residual hearing after cochlear implant surgery with slim modiolar electrode. *Eur. Arch. Otorhinolaryngol.* 277 367–375. 10.1007/s00405-019-05708-x 31673779 PMC6981311

[B33] James C. Albegger K. Battmer R. Burdo S. Deggouj N. Deguine O. (2005). Preservation of residual hearing with cochlear implantation: how and why. *Acta Otolaryngol.* 125 481–491. 10.1080/00016480510026197 16092537

[B34] Jayakody D. M. P. Friedland P. L. Nel E. Martins R. N. Atlas M. D. Sohrabi H. R. (2017). Impact of cochlear implantation on cognitive functions of older adults: pilot test results. *Otol. Neurotol.* 38 e289–e295. 28806341 10.1097/MAO.0000000000001502

[B35] Jurawitz M. C. Büchner A. Harpel T. Schüssler M. Majdani O. Lesinski-Schiedat A. (2014). Hearing preservation outcomes with different cochlear implant electrodes: nucleus^®^ Hybrid™-L24 and Nucleus Freedom™ CI422. *Audiol. Neurootol.* 19 293–309. 10.1159/000360601 25277083

[B36] Knopke S. Häussler S. Gräbel S. Wetterauer D. Ketterer M. Fluger A. (2019). Age-Dependent Psychological Factors Influencing the Outcome of Cochlear Implantation in Elderly Patients. *Otol. Neurotol.* 40 e441–e453. 10.1097/MAO.0000000000002179 30870379

[B37] Korkmaz M. H. Bayır Ö Er S. Işık E. Saylam G. Tatar E. Ç (2016). Satisfaction and compliance of adult patients using hearing aid and evaluation of factors affecting them. *Eur. Arch. Otorhinolaryngol.* 273 3723–3732. 10.1007/s00405-016-4046-x 27094053

[B38] Labadie R. F. Carrasco V. N. Gilmer C. H. Pillsbury H. C. (2000). Cochlear implant performance in senior citizens. *Otolaryngol. Head Neck Surg.* 123 419–424. 10.1067/mhn.2000.109759 11020178

[B39] Lenarz M. Sönmez H. Joseph G. Büchner A. Lenarz T. (2012). Cochlear implant performance in geriatric patients. *Laryngoscope* 122 1361–1365. 10.1002/lary.23232 22539093

[B40] Lenarz T. Stöver T. Buechner A. Lesinski-Schiedat A. Patrick J. Pesch J. (2009). Hearing Conservation Surgery Using the Hybrid-L Electrode. Results from the First Clinical Trial at the Medical University of Hannover. *Audiol. Neurotol.* 14 22–31. 10.1159/000206492 19390172

[B41] Lenarz T. Timm M. E. Salcher R. Büchner A. (2019). Individual Hearing Preservation Cochlear Implantation Using the Concept of Partial Insertion. *Otol. Neurotol.* 40 e326–e335. 10.1097/MAO.0000000000002127 30741914

[B42] Li C. M. Zhang X. Hoffman H. J. Cotch M. F. Themann C. L. Wilson M. R. (2014). Hearing Impairment Associated With Depression in US Adults, National Health and Nutrition Examination Survey 2005-2010. *JAMA Otolaryngol. Head Neck Surg.* 140 293–302. 10.1001/jamaoto.2014.42 24604103 PMC4102382

[B43] Livingston G. Huntley J. Sommerlad A. Ames D. Ballard C. Banerjee S. (2020). Dementia prevention, intervention, and care: 2020 report of the Lancet Commission. *Lancet* 396 413–446. 10.1016/S0140-6736(20)30367-6 32738937 PMC7392084

[B44] Matin F. Artukarslan E. N. Illg A. Lesinski-Schiedat A. Lenarz T. Suhling M. C. (2021). Cochlear Implantation in Elderly Patients with Residual Hearing. *J. Clin. Med.* 10:4305. 10.3390/jcm10194305 34640325 PMC8509733

[B45] Mosnier I. Bebear J. P. Marx M. Fraysse B. Truy E. Lina-Granade G. (2015). Improvement of Cognitive Function After Cochlear Implantation in Elderly Patients. *JAMA Otolaryngol. Head Neck Surg.* 141 442–450. 25763680 10.1001/jamaoto.2015.129

[B46] Olze H. Szczepek A. J. Haupt H. Förster U. Zirke N. Gräbel S. (2011). Cochlear Implantation Has a Positive Influence on Quality of Life, Tinnitus, and Psychological Comorbidity. *Laryngoscope* 121 2220–2227. 10.1002/lary.22145 21898434

[B47] Parker M. J. Handoll H. H. G. Griffiths R. (2004). Anaesthesia for hip fracture surgery in adults. *Cochrane Database Syst. Rev.* 2:CD000521. 10.1002/14651858.CD000521.pub2 15494999

[B48] Pasanisi E. Bacciu A. Vincenti V. Guida M. Barbot A. Berghenti M. T. (2003). Speech recognition in elderly cochlear implant recipients. *Clin. Otolaryngol. Allied Sci.* 28 154–157. 12680835 10.1046/j.1365-2273.2003.00681.x

[B49] Roth T. N. Hanebuth D. Probst R. (2011). Prevalence of age-related hearing loss in Europe: a review. *Eur. Arch. Otorhinolaryngol.* 268 1101–1107. 10.1007/s00405-011-1597-8 21499871 PMC3132411

[B50] Rutherford B. R. Brewster K. Golub J. S. Kim A. H. Roose S. P. (2018). Sensation and Psychiatry: linking Age-Related Hearing Loss to Late-Life Depression and Cognitive Decline. *Am. J. Psychiatry* 175 215–224. 10.1176/appi.ajp.2017.17040423 29202654 PMC5849471

[B51] Sarant J. Harris D. Busby P. Maruff P. Schembri A. Dowell R. (2019). The Effect of Cochlear Implants on Cognitive Function in Older Adults: initial Baseline and 18-Month Follow Up Results for a Prospective International Longitudinal Study. *Front. Neurosci.* 2:789. 10.3389/fnins.2019.00789 31427915 PMC6687844

[B52] Singh N. K. Jha R. H. Gargeshwari A. Kumar P. (2018). Altered auditory and vestibular functioning in individuals with low bone mineral density: a systematic review. *Eur. Arch. Otorhinolaryngol.* 275 1–10. 10.1007/s00405-017-4768-4 29043479

[B53] Skarzynski H. Lorens A. Piotrowska A. Anderson I. (2007). Partial deafness cochlear implantation in children. *Int. J. Pediatr. Otorhinolaryngol.* 71 1407–1413. 17597232 10.1016/j.ijporl.2007.05.014

[B54] Smulders Y. E. Hendriks T. Eikelboom R. H. Stegeman I. Santa Maria P. L. Atlas M. D. (2017). Predicting Sequential Cochlear Implantation Performance: a Systematic Review. *Audiol. Neurootol.* 22 356–363. 10.1159/000488386 29719297

[B55] Sonnet M. H. Montaut-Verient B. Niemier J. Y. Hoen M. Ribeyre L. Parietti-Winkler C. (2017). Cognitive Abilities and Quality of Life After Cochlear Implantation in the Elderly. *Otol. Neurotol.* 38 e296–e301. 10.1097/MAO.0000000000001503 28806342

[B56] Steffens T. Müller-Deile J. Kiessling J. (2013). Auch im Alter noch gut verstehen mit Cochlea-Implantaten. Sprachverstehen bei altersbedingter Schwerhörigkeit. *HNO Nachrichten* 43 17–22.

[B57] Sterkers O. Mosnier I. Ambert-Dahan E. Herelle-Dupuy E. Bozorg-Grayeli A. Bouccara D. (2004). Cochlear Implants in elderly people: preliminary results. *Acta Otolaryngol.* 124 64–67. 26942828 10.1080/03655230410017184

[B58] Suhling M. C. Majdani O. Salcher R. Leifholz M. Büchner A. Lesinski-Schiedat A. (2016). The Impact of Electrode Array Length on Hearing Preservation in Cochlear Implantation. *Otol. Neurotol.* 37 1006–1015. 10.1097/MAO.0000000000001110 27309713

[B59] Sun Z. Seo J. W. Park H. J. Lee J. Y. Kwak M. Y. Kim Y. (2021). Cortical reorganization following auditory deprivation predicts cochlear implant performance in postlingually deaf adults. *Hum. Brain Mapp.* 42 233–244. 10.1002/hbm.25219 33022826 PMC7721232

[B60] United Nations (2019). *Department of Economic and Social Affairs, Population Division. World Population Ageing 2019.* New York: United Nations

[B61] Urwin S. C. Parker M. J. Griffiths R. (2000). General versus regional anaesthesia for hip fracture surgery: a meta-analysis of randomized trials. *Br. J. Anaesth.* 84 450–455. 10823094 10.1093/oxfordjournals.bja.a013468

[B62] Völter C. Götze L. Dazert S. Falkenstein M. Thomas J. P. (2018). Can cochlear implantation improve neurocognition in the aging population. *Clin. Interv. Aging* 13 701–712. 10.2147/CIA.S160517 29719382 PMC5916259

[B63] von Ilberg C. A. Baumann U. Kiefer J. Tillein J. Adunka O. F. (2011). Electric-acoustic stimulation of the auditory system: a review of the first decade. *Audiol. Neurootol.* 16 1–30. 10.1159/000327765 21606646

[B64] Wilkerson B. J. Porps S. F. Babu S. C. (2017). The Impact of Comorbidities in the Aging Population on Cochlear Implant Outcomes. *Otol. Neurotol.* 38 e285–e288. 10.1097/MAO.0000000000001501 28806340

[B65] Williamson R. A. Pytynia K. Oghalai J. S. Vrabec J. T. (2009). Auditory performance after cochlear implantation in late septuagenarians and octogenarians. *Otol. Neurotol.* 30 298–301. 10.1097/MAO.0b013e3181b4e594 19672204 PMC3607509

[B66] World Health Organization (2021). *World Report on Hearing.* Geneva: World Health Organization

